# Herpes Simplex Virus 1 Lytic Infection Blocks MicroRNA (miRNA) Biogenesis at the Stage of Nuclear Export of Pre-miRNAs

**DOI:** 10.1128/mBio.02856-18

**Published:** 2019-02-12

**Authors:** Dongli Pan, Gang Li, Jenna Morris-Love, Shuyuan Qi, Lei Feng, Max E. Mertens, Igor Jurak, David M. Knipe, Donald M. Coen

**Affiliations:** a Department of Medical Microbiology and Parasitology, Zhejiang University School of Medicine, Hangzhou, Zhejiang, China; b Department of Infectious Diseases of Sir Run Run Shaw Hospital, Zhejiang University School of Medicine, Hangzhou, Zhejiang, China; c Department of Biological Chemistry and Molecular Pharmacology, Harvard Medical School, Boston, Massachusetts, USA; d Department of Biotechnology, University of Rijeka, Rijeka, Croatia; e Department of Microbiology, Harvard Medical School, Boston, Massachusetts, USA; f Program in Virology, Harvard Medical School, Boston, Massachusetts, USA; Virginia Polytechnic Institute and State University; University of California, Irvine; University of North Carolina at Chapel Hill

**Keywords:** herpes simplex virus, ICP27, latency, miRNAs, nuclear export

## Abstract

Herpes simplex virus 1 (HSV-1) switches between two infection programs, productive (“lytic”) and latent infection. Some HSV-1 microRNAs (miRNAs) have been hypothesized to help control this switch, and yet little is known about regulation of their expression. Using Northern blot analyses, we found that, despite inherent differences in biogenesis efficiency among six HSV-1 miRNAs, all six exhibited high pre-miRNA/miRNA ratios during lytic infection of different cell lines and, when detectable, in acutely infected mouse trigeminal ganglia. In contrast, considerably lower ratios were observed in latently infected ganglia and in cells transduced with lentiviral vectors expressing the miRNAs, suggesting that HSV-1 lytic infection blocks miRNA biogenesis. This phenomenon is not specific to viral miRNAs, as a host miRNA expressed from recombinant HSV-1 also exhibited high pre-miRNA/miRNA ratios late during lytic infection. The levels of most of the mature miRNAs remained stable during infection in the presence of actinomycin D, indicating that the high ratios are due to inefficient pre-miRNA conversion to miRNA. Cellular fractionation experiments showed that late (but not early) during infection, pre-miRNAs were enriched in the nucleus and depleted in the cytoplasm, indicating that nuclear export was blocked. A mutation eliminating ICP27 expression or addition of acyclovir reduced pre-miRNA/miRNA ratios, but mutations drastically reducing Us11 expression did not. Thus, HSV-1 lytic infection inhibits miRNA biogenesis at the step of nuclear export and does so in an ICP27- and viral DNA synthesis-dependent manner. This mechanism may benefit the virus by reducing expression of repressive miRNAs during lytic infection while permitting elevated expression during latency.

## Abstract (precis)

Various mechanisms have been identified by which viruses target host small RNA biogenesis pathways to achieve optimal infection outcomes. Herpes simplex virus 1 (HSV-1) is a ubiquitous human pathogen whose successful persistence in the host entails both productive (“lytic”) and latent infection. Although many HSV-1 miRNAs have been discovered and some are thought to help control the lytic/latent switch, little is known about regulation of their biogenesis. By characterizing expression of both pre-miRNAs and mature miRNAs under various conditions, this study revealed striking differences in miRNA biogenesis between lytic and latent infection and uncovered a regulatory mechanism that blocks pre-miRNA nuclear export and is dependent on viral protein ICP27 and viral DNA synthesis. This mechanism represents a new virus-host interaction that could limit the repressive effects of HSV-1 miRNAs hypothesized to promote latency and may shed light on the regulation of miRNA nuclear export, which has been relatively unexplored.

## INTRODUCTION

Herpes simplex virus 1 (HSV-1) is a prevalent human pathogen that establishes lifelong latency in sensory neurons following productive (“lytic”) infection in peripheral tissues (reviewed in reference 1). HSV-1 encodes at least 21 microRNAs (miRNAs) (2–8), many of which are conserved in HSV-2 (4, 9–11). Some miRNAs, together with the ∼2-kb-intron latency-associated transcripts (LAT), are the only viral gene products known to be highly expressed during latency (12). Hypothesized functions of HSV-1 miRNAs include repression of lytic gene expression during latency (3, 13–15), repression of viral replication (5, 16), antagonism of intrinsic and innate immunity (6, 17, 18), and promotion of viral replication (7). Because miRNAs function efficiently only when they are abundant (19), it is important to understand regulation of miRNA expression.

miRNAs are derived from transcripts (pri-miRNAs) with embedded stem-loops. Typically, Drosha, a nuclear RNase, cleaves pri-miRNAs into smaller hairpin-shaped RNAs known as pre-miRNAs. Exportin 5 then binds pre-miRNAs in a Ran-GTP-dependent manner and exports them into the cytoplasm, where they are further cleaved by another RNase, Dicer, releasing small RNA duplexes. One strand of each duplex is subsequently loaded into an RNA-induced silencing complex containing the Argonaute (Ago) protein, which targets the complex to complementary mRNAs (reviewed in references 19 and 20).

HSV-1 lytic infection and latent infection display different viral miRNA expression profiles. During lytic infection, miR-H1 and miR-H6 appear to be the most abundantly expressed (4, 12, 13). However, during latency, miR-H1 is poorly expressed whereas miR-H2, miR-H4-5p, miR-H4-3p, and miR-H5 to miR-H8 are abundant (8, 12, 21–23). Except for miR-H6, these miRNAs are located within the *LAT* transcription unit, suggesting that they are cleavage products of the primary 8.3-kb *LAT*.

Most of our knowledge about HSV-1 miRNA expression has come from deep sequencing and stem-loop reverse transcription-quantitative PCR (qRT-PCR) analyses, which provide no information regarding miRNA precursors. In contrast, Northern blot hybridization (Northern) permits detection of both pre-miRNA and mature miRNAs with high specificity. Starting with the initial reports of HSV miRNAs (2, 3, 9, 10, 12, 15, 24, 25), Northern results indicated an interesting phenomenon of high expression of viral pre-miRNAs but low expression of their mature miRNAs during lytic infection. Specifically, for HSV-1, pre-miR-H1 to pre-miR-H6 are detected readily, but their mature counterparts are detected only weakly if at all (2, 3, 12, 15), in contrast to what is seen with most cellular miRNAs, such as let-7a (12). This is also true for HSV-2 miRNAs analyzed by Northern (9, 10, 24, 25). This mechanism of limited miRNA expression despite high pre-miRNA expression might operate to restrict the functions of these miRNAs during lytic infection.

To understand this phenomenon, we conducted a series of Northern-based experiments. The results not only revealed distinct HSV-1 miRNA expression patterns in cell culture and mouse ganglia but also uncovered a regulatory mechanism that inhibits pre-miRNA nuclear export.

## RESULTS

### High ratios of HSV-1 pre-miRNAs to miRNAs during lytic infection.

**FIG 1:**
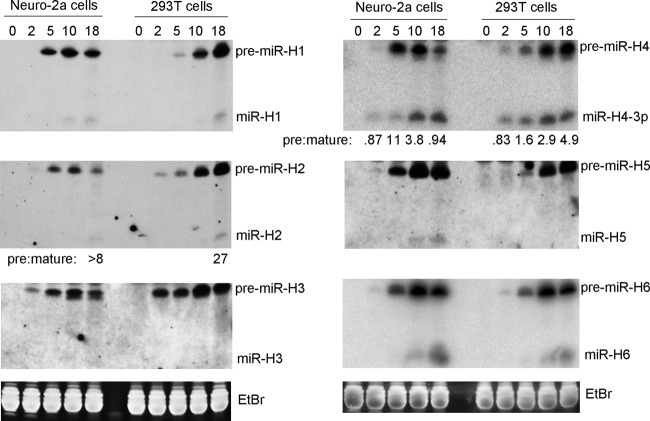
HSV-1 pre-miRNA and mature miRNA expression in Neuro-2a and 293T cells. Neuro-2a and 293T cells were infected with HSV-1 strain KOS at MOIs of 30 and 10, respectively. At various times postinfection, cells were harvested and small RNAs (<200 bases) were purified. Following polyacrylamide gel electrophoresis, RNAs were transferred to membranes and probed for miRNA expression by Northern blot hybridization. Cell line names and times of cell harvest (in h) are indicated at the top of the Northern images. Positions of miRNA species are labeled to the right of the images. At the bottom are ethidium bromide (EtBr) staining signals from the gels at ∼80 bases showing uniform loading for the images above them. Pre/mature ratios calculated following band quantification are shown below the images for miR-H2 and miR-H4. This experiment was performed twice. Similar results were also obtained for the specific time points indicated in Fig. 2 (see also Fig. S1).

Because Northern analyses had been performed previously for HSV-1 miRNAs only in African green monkey kidney (Vero) cells, we analyzed expression of miR-H1 to miR-H6 using two additional cell lines, human embryonic kidney (293T) cells and mouse neuroblastoma (Neuro-2a) cells, following infection at multiplicities of infection (MOI) of 10 and 30, respectively, by HSV-1 wild-type (WT) strain KOS. These miRNAs (and the corresponding pre-miRNAs) are expressed as late gene products in Vero cells from unknown promoters (deletion of the *LAT* promoter does not affect their expression during lytic infection as it does during latency) (12). In addition to species detected by previous Northern analyses in Vero cells, including pre-miR-H1 to pre-miR-H6 and mature miR-H1, miR-H2, and miR-H6, we additionally detected mature miR-H4-3p in both 293T and Neuro-2a cells and miR-H5 in Neuro-2a cells (Fig. 1). All 6 pre-miRNAs became detectable by 5 h postinfection (hpi) and abundant at 10 and 18 hpi in both cell lines. However, mature miR-H3 was not detected in either cell line, consistent with its very low level of expression (<1 molecule/cell) as measured by qRT-PCR of <40-nucleotide (nt) RNA in Vero cells (12). Mature miR-H1, miR-H2, miR-H5, and miR-H6 were detectable at late times and at much lower abundances than their precursors. miR-H4 was detectable as early as 2 hpi, as previously observed by qRT-PCR in Vero cells (12), and eventually reached a level that was similar to that seen with pre-miR-H4 in Neuro-2a cells but still substantially less than that of pre-miR-H4 in 293T cells. Taking the results together, despite slight differences observed among different miRNAs and cell lines, there were generally high pre-miRNA/mature miRNA (pre/mature) ratios in both 293T and Neuro-2a cells, as shown here, and in Vero cells, as shown previously (2, 4, 12). Thus, this phenomenon was observed in multiple species and cell types.

10.1128/mBio.02856-18.1 FIG S1 HSV-1 pre-miRNA and mature miRNA expression in trigeminal ganglia at different times after infection or after reactivation from latency. Mice were mock infected or infected on the cornea with 2 × 10^5^ PFU of HSV-1 strain KOS per eye. At various days postinfection (dpi), trigeminal ganglia were collected. At 30 dpi, some ganglia were explanted and cultivated for various days postexplantation (dpe) before the ganglia were harvested for RNA. Sixteen ganglia were pooled for each condition, and small RNAs (<200 bases) were isolated and analyzed by Northern blot hybridization. RNAs purified from 293T cells that had been mock infected or infected for 18 h were run alongside (see last two lanes) the RNAs from ganglia to provide markers. The sources of RNAs and time points are indicated at the tops of each series (left or right) of Northern images. Positions of miRNA species are indicated to the right of the images. Ethidium bromide signals at ∼80 bases from the gels used for each series of Northern images are displayed at the bottom. An independent experiment is shown in Fig. 1. Download FIG S1, PDF file, 1.3 MB. Copyright © 2019 Pan et al. 2019 Pan et al. This content is distributed under the terms of the Creative Commons Attribution 4.0 International license.

### Differential miRNA biogenesis in acutely and latently infected mouse trigeminal ganglia.

**FIG 2:**
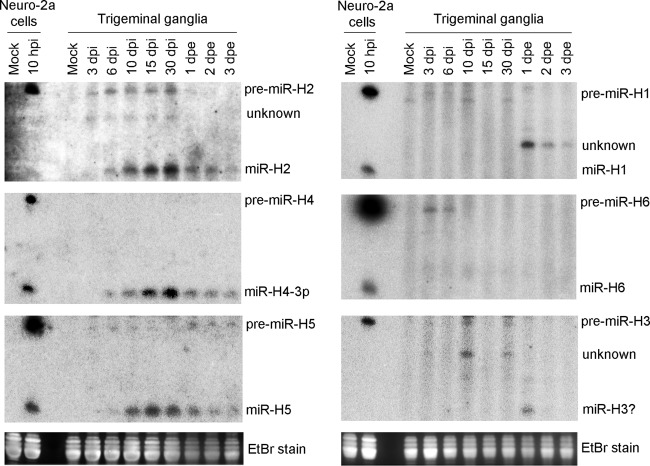
HSV-1 pre-miRNA and mature miRNA expression in trigeminal ganglia at different times after infection or after reactivation from latency. Mice were mock infected or infected on the cornea with 2 × 10^5^ PFU of HSV-1 strain KOS per eye. At the various days postinfection (dpi) indicated, trigeminal ganglia were collected for immediate RNA isolation. Additionally, at 30 dpi, some ganglia were explanted and cultivated for various days postexplantation (dpe) before RNA was isolated. Sixteen ganglia were pooled for each condition, and small RNAs (<200 bases) were isolated and analyzed by Northern blot hybridization. RNAs purified from Neuro-2a cells that had been mock infected or infected with HSV-1 strain KOS for 10 h were run alongside (first two lanes) the RNAs from ganglia to provide markers. The sources of RNAs and time points are indicated at the tops of each series (left and right, respectively) of Northern images. Positions of miRNA species are indicated to the right of the images. Ethidium bromide signals at ∼80 bases from the gels used for each series of Northern images are displayed at the bottom. Results of an independent experiment are shown in Fig. S1.

To investigate whether high pre/mature ratios also occur *in vivo*, we performed two independent experiments (Fig. 2; see also Fig. S1 in the supplemental material) in which we infected mice on the cornea with 2 × 10^5^ PFU/eye of HSV-1 and analyzed miRNA expression in trigeminal ganglia on various days postinfection (dpi) by Northern blotting. We also explanted some ganglia at 30 dpi and cultivated them for 1 to 3 days to reactivate the virus before collecting the tissues for RNA analysis. miR-H2, miR-H4, and miR-H5 showed one expression pattern: Their mature miRNAs were readily detected after 6 dpi, with levels steadily increasing through 30 dpi and then decreasing during reactivation (Fig. 2; see also Fig. S1). This is consistent with previous qRT-PCR data (12, 22, 26). However, during establishment and maintenance of latency (6 to 30 dpi), pre-miR-H4 was undetectable and pre-miR-H2 and H5 were much less abundant than their mature forms (Fig. 2; see also Fig. S1). The miRNAs that are most abundant during lytic infection, miR-H1 and miR-H6, showed a different pattern. At the height of acute ganglionic infection (3 dpi), pre-miR-H1, but not miR-H1, was detected weakly, and neither was detected subsequently (Fig. 2; see also Fig. S1), consistent with previous qRT-PCR results (4, 12, 22). Pre-miR-H6, but not mature miR-H6, was clearly detectable at 3 and 6 dpi, but neither pre-miR-H6 nor mature miR-H6 was detected later (Fig. 2; see also Fig. S1). Our inability to detect miR-H6 was not consistent with previous qRT-PCR results that showed high miR-H6 expression levels in both acutely infected ganglia and latently infected ganglia (3, 12, 22). Insufficient sensitivity of our Northern analyses might explain this discrepancy. Finally, we did not reproducibly detect bands corresponding to pre-miR-H3 or mature miR-H3 that were clearly distinct from background in mouse ganglia, which was consistent with undetectable levels in previous qRT-PCR results obtained from <40-nt RNA (12) but not with reported high levels in unfractionated RNA (22). We suspect that the latter result was due to the presence of longer species contributing to miR-H3 qRT-PCR signals. Taking the results together, during establishment and maintenance of latency, mature miRNAs, whenever detectable, were always more abundant than pre-miRNAs (i.e., low pre/mature ratios), whereas at the height of acute infection (3 dpi), no mature miRNA was consistently detected despite multiple pre-miRNAs being detected (high pre/mature ratios), highlighting the contrast between acute infection and latent infection in miRNA biogenesis.

### Lower pre/mature ratios in lentivirus-transduced cells than during HSV-1 lytic infection.

**FIG 3:**
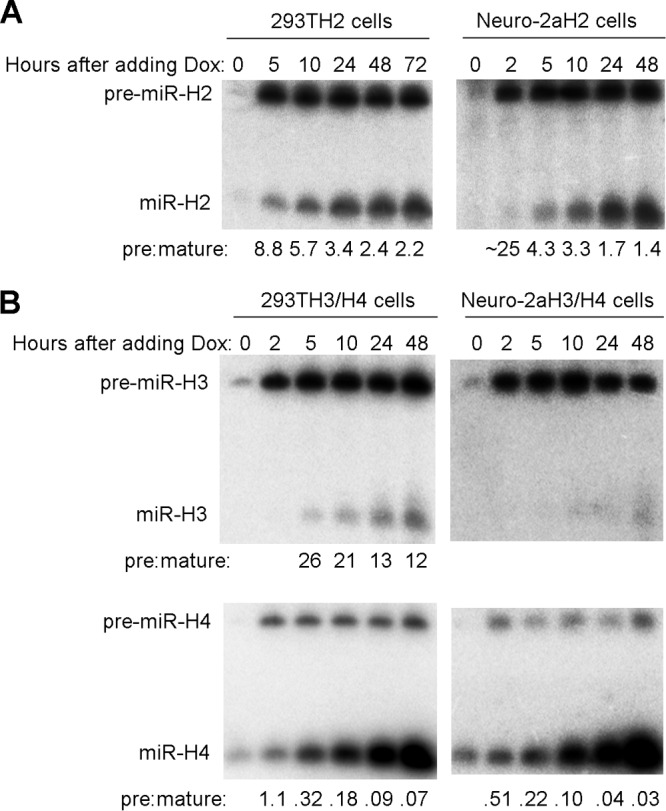
Conversion of pre-miRNA to mature miRNA during induced expression following lentiviral transduction. (A) 293T and Neuro-2a cells were stably transduced with an inducible pre-miR-H2 expressing lentivirus, resulting in 293TH2 and Neuro-2aH2 cell lines. After 1 μg/ml of Dox was added, cells were harvested at different times and analyzed by Northern blot hybridization for miR-H2 expression. Cell line names and time points are indicated at the tops of the images. Positions of miRNA species are labeled to the left. Ratios of band intensities of pre-miRNAs to those of miRNAs are shown at the bottom. The experiment using 293TH2 cells was performed twice; the experiment using Neuro-2aH2 cells was performed once. (B) As described for panel A, but the 293TH3/H4 and Neuro-2aH3/H4 cell lines were created by transduction using a lentivirus expressing miR-H3 and miR-H4, and miR-H3 and H4 expression levels were analyzed. These experiments were repeated with cells constructed using different lentivirus constructs expressing miR-H3 and/or miR-H4.

We next asked whether the high pre/mature ratios seen during lytic infection might have been due simply to a lack of sufficient time to complete biogenesis. To determine how quickly mature miRNAs could be expressed in cells, we employed an inducible lentiviral transduction system. We cloned sequences encoding miR-H2 or miR-H3/H4 pre-miRNAs along with flanking sequences into the pTRIPZ vector, in which inducible gene expression is driven by a tetracycline-responsive promoter (sequences encoding pre-miR-H3 and -H4 were cloned as one fragment as they are separated by only 129 bp). We then produced lentiviruses and used them to stably transduce 293T and Neuro-2a cells. We monitored miRNA expression following doxycycline (Dox) addition to the transduced cells. The three miRNAs differed greatly in their final pre/mature ratios, with miR-H4 having the lowest and miR-H3 the highest in both the 293T-derived and Neuro-2a-derived cell lines (Fig. 3), suggesting important inherent differences in biogenesis efficiency among them. Nevertheless, the transduced cells always exhibited more rapid and efficient miRNA biogenesis than that seen in HSV-1-infected cells (Fig. 1). For example, the miR-H2 and miR-H4 pre/mature ratios seen at 5 h after addition of Dox were already lower than those seen at 18 h after HSV-1 infection, and mature miR-H3 was detectable in transduced cells but undetectable in infected cells despite the high pre-miR-H3 levels in both settings. These results argue for a regulatory mechanism impeding accumulation of mature miRNAs during lytic infection.

### High pre/mature ratios are due to inefficient pre-miRNA conversion to mature miRNA rather than to mature miRNA destabilization.

**FIG 4:**
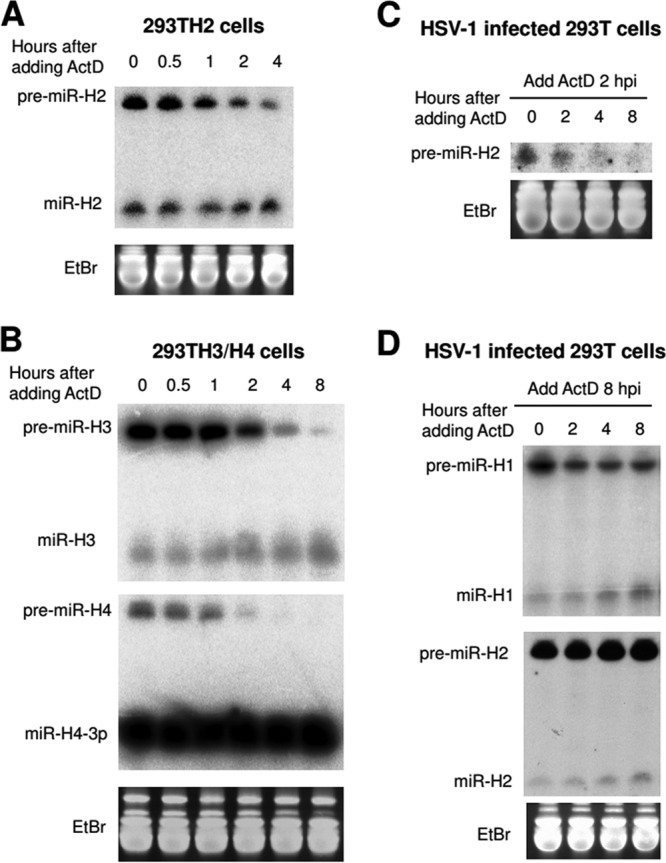
Stability of pre-miRNAs and miRNAs in lentivirus-transduced cells and HSV-1-infected cells. (A) miR-H2 expression from 293TH2 cells was induced by adding 1 μg/ml of Dox for 3 days. Then, 1 μg/ml of ActD was added to inhibit transcription. Cells were harvested at the times indicated at the top of the image and were analyzed by Northern blot hybridization for miR-H2 expression. Uniform loading on the gel used for the Northern blot hybridization was demonstrated by ethidium bromide staining (shown at the bottom). This experiment was performed once. (B) As described for panel A, but 293TH3/H4 cells were used and the levels of expression of miR-H3 (upper panel) and miR-H4 (lower panel) were analyzed. This experiment was repeated with cells constructed using different lentivirus vectors expressing miR-H3 and/or miR-H4. (C) 293T cells were infected with HSV-1 strain KOS (MOI = 10). At 2 hpi, 1 μg/ml ActD was added, and cells were harvested at the times indicated at the top of the images and analyzed by Northern blot hybridization for miR-H2 expression. The upper image shows pre-miR-H2 expression (mature miR-H2 was not detectable at that time point). The bottom images show the ethidium bromide-stained gel at ∼80 bases. (D) As described for panel C, but ActD was added at 8 hpi, and miR-H1 (upper panel) and miR-H2 (lower panel) were analyzed. Positions of miRNA species are indicated to the left. The experiments shown in panels C and D were performed twice; additionally, results for miR-H4 are shown in Fig. S2.

Two hypotheses could explain the high pre/mature miRNA ratios: (i) block of pre-to-mature conversion and (ii) expedited degradation of mature miRNA. To investigate these hypotheses, we analyzed miRNA stability following addition of a transcription inhibitor, actinomycin D (ActD). In transduced 293T cells, at 3 days following Dox addition, mature miR-H2, miR-H3, and miR-H4 were all stable for 8 h following ActD addition, but the corresponding pre-miRNAs were all much less stable (Fig. 4A and B). In HSV-1-infected cells, when ActD was added at 2 hpi, pre-miR-H2 and pre-miR-H4 levels decreased substantially over the next 8 h (Fig. 4C; see also Fig. S2). However, when ActD was added at 8 hpi, pre-miR-H2 levels did not decrease over the 8 h that followed, and pre-miR-H4 and pre-miR-H1 levels decreased by only ∼2-fold during this time frame (Fig. 4D; see also Fig. S2). Following ActD addition at either 2 or 8 hpi, the levels of the corresponding mature miRNAs remained stable or even increased, with the possible exception of the level of miR-H4, which appeared to decrease and then become stable following ActD addition at 8 hpi (Fig. S2). These results suggest that inefficient conversion of pre-miRNA to mature miRNA, rather than mature miRNA instability, accounts for high pre/mature ratios and that pre-miRNAs become more stable over the course of infection, consistent with interference with pre-miRNA conversion to miRNA late in infection.

10.1128/mBio.02856-18.2 FIG S2 Stability of pre-miRNAs and miRNAs in HSV-1-infected cells. 293T cells were infected with HSV-1 strain KOS (MOI = 10). At 2 (left panel) or 8 (right panel) hpi, 1 μg/ml ActD was added, and cells were harvested at the times indicated at the top of the images. miR-H4 was analyzed by Northern blot hybridization. Results of experiments examining miR-H2 are shown in Fig. 4C and D. Download FIG S2, PDF file, 0.4 MB. Copyright © 2019 Pan et al. 2019 Pan et al. This content is distributed under the terms of the Creative Commons Attribution 4.0 International license.

### A host miRNA expressed from a recombinant HSV-1 exhibited inefficient pre-miRNA processing during lytic infection.

**FIG 5:**
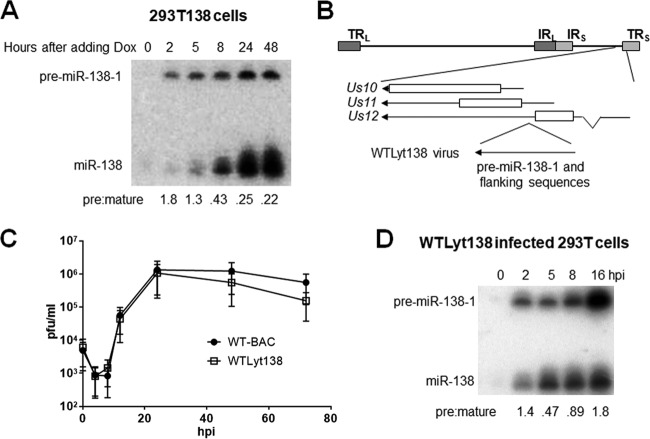
miR-138 expression in lentivirus-transduced cells and in cells infected with recombinant HSV-1 expressing miR-138. (A) 293T cells were stably transduced with an inducible pre-miR-138-1-expressing lentivirus, resulting in the 293T138 cell line. After 1 μg/ml of Dox was added, cells were harvested at the times indicated at the top of the image and analyzed by Northern blot hybridization for miR-138 expression. Pre/mature ratios calculated following band quantification are shown at the bottom. This experiment was performed twice. (B) Genomic location of inserted pre-miR-138-1-expressing sequences. The HSV-1 genome is depicted as a horizontal line at the top with long repeat sequences (TR_L_ and IR_L_) and short repeat sequences (IR_S_ and TR_S_), which are shown as dark and light gray boxes, respectively. An expanded view of the insertion location is shown below the horizontal line, with bars representing protein coding sequences, arrows representing transcripts and gene names provided to the left. As shown at the bottom, pre-miR-138-1 and flanking sequences were inserted between coding sequences of *Us11* and *Us12*, resulting in a recombinant virus designated WTLyt138. (C) Replication kinetics in Vero cells (MOI = 0.02). Each point represents the average of titers from three biological replicates (the experiment was performed three times), and the vertical bars represent standard deviations. (D) 293T cells were infected with WTLyt138 (MOI = 5). The image shows an autoradiogram of the Northern blot of RNAs isolated from the cells at the hours postinfection (hpi) indicated at the top of the panel, hybridized for miR-138. Positions of pre-miR-138-1 and miR-138 are indicated to the left. Pre/mature ratios calculated following band quantification are shown at the bottom. This experiment was performed three times; additionally, similar results were obtained at two time points in Fig. 7F.

We wondered whether this impairment of biogenesis is specific to viral miRNAs. The effects of HSV infection on endogenous or transduced miRNAs are difficult to study due to the already high levels of most mature host miRNAs and the shutoff of most host transcription (reviewed in reference 27). To avoid these issues, we expressed miR-138 as an HSV-1 gene product. This miRNA was chosen because its low level of endogenous expression in 293T cells (28) allows easy detection of its exogenous expression from a virus. In an initial experiment, we cloned pre-miR-138-1 and flanking sequences into the inducible lentiviral transduction system described above to assess the intrinsic biogenesis efficiency of this miRNA. Upon Dox addition to miR-138-expressing 293T cells, pre-miR-138-1 levels were nearly constant from 2 to 48 h, but mature miR-138 levels increased steadily, resulting in pre/mature ratios of about 0.25 by 24 h (Fig. 5A), indicating that the biogenesis of miR-138 is intrinsically efficient. We then modified the HSV-1 genome to insert the same pre-miR138-1 and flanking sequences between the *Us11* and *Us12* open reading frames such that miR-138 would be encoded within the *Us12* 3′ untranslated region (UTR) and *Us11* 5′ UTR and thus would be expressed from both the *Us12* immediate early and *Us11* late promoters (Fig. 5B). The resulting recombinant HSV-1, designated WTLyt138, showed replication kinetics in Vero cells indistinguishable from those of the corresponding bacterial artificial chromosome (BAC)-derived WT parental virus (WT-BAC) (Fig. 5C) and showed no more than a modest defect in Us11 expression (data not shown) which should not affect miRNA biogenesis, because deletion of *Us11* has little if any impact on miRNA biogenesis (see below). As expected for transcription from both immediate early and late promoters, pre-miR-138-1 expression from WTLyt138 virus appeared to be biphasic, with initial expression at 2 hpi followed by a decrease in the level of expression at 5 hpi and then an increase again after 5 hpi (Fig. 5D). Pre-miRNA conversion to mature miRNA was efficient during the first phase, with a pre/mature ratio of 0.47 at 5 hpi. After 5 hpi, however, mature miR-138 levels increased only slightly despite dramatic increases in pre-miR-138-1 levels, resulting in a 4-fold-higher pre/mature ratio at 16 hpi than at 5 hpi, arguing that biogenesis of a host miRNA was also impeded late during lytic infection.

### Pre-miRNA nuclear export is blocked during HSV-1 lytic infection.

To understand the mechanism of this inefficient biogenesis, we first asked whether infection changed levels of key factors involved in miRNA biogenesis and found that Drosha, Exportin 5, and Dicer were not downregulated during HSV-1 infection (Fig. S3), indicating that changes in the expression levels of these proteins did not explain inefficient miRNA biogenesis.

10.1128/mBio.02856-18.3 FIG S3 HSV-1 infection does not reduce expression of various proteins involved in miRNA biogenesis. 293T and Vero cells were infected at an MOI of 10 and Neuro-2a cells were infected at an MOI of 30 with HSV-1 strain KOS for the times indicated at the top of the panel. The proteins indicated to the left were analyzed by Western blotting. The experiment using Vero and 293T cells was performed multiple times. The experiment using Neuro-2a cells was performed once. Download FIG S3, PDF file, 0.2 MB. Copyright © 2019 Pan et al. 2019 Pan et al. This content is distributed under the terms of the Creative Commons Attribution 4.0 International license.

**FIG 6:**
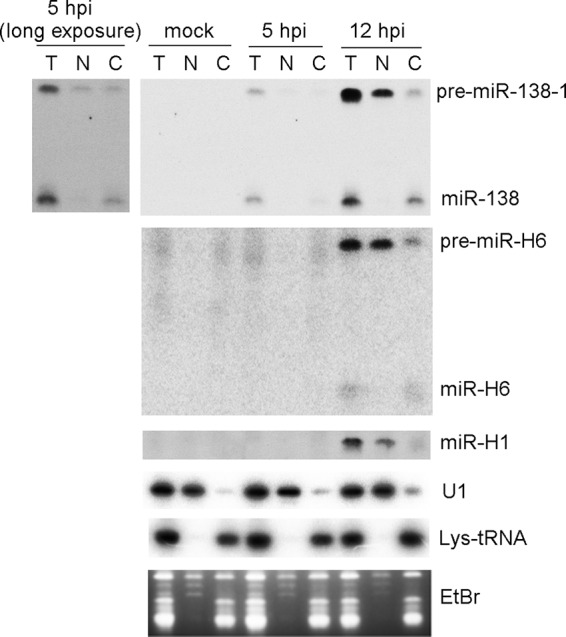
Nuclear export of pre-miRNA is blocked at late times during HSV-1 lytic infection. 293T cells were mock infected or infected with WTLyt138 virus (MOI = 5) for 5 or 12 h, at which times cells were harvested either for direct RNA purification (total) or for separation into nuclear and cytoplasmic fractions prior to RNA purification. The resulting RNAs were subjected to Northern blot hybridization for analysis of the RNAs indicated to the right of the panels. Mock infection and times postinfection (pi) are indicated at the top of the panel. The fractions are identified above the images. T, total RNA; N, nuclear fraction; C, cytoplasmic fraction. Mature miR-H1 was not detected in this experiment. The gel stained with ethidium bromide at ∼80 to ∼110 bases is shown in the bottom panel. An image from a longer exposure of the miR-138 signals is shown to the left of the main panel for miR-138. This experiment was performed twice. Additionally, similar results were obtained for miR-H6 in KOS-infected or KOS1.1-infected cells (Fig. 7E; see also Fig. S4).

We then analyzed miRNA expression in nuclear and cytoplasmic fractions of WTLyt138-infected cells. The biphasic expression of miR-138 from this virus facilitated analysis of this experiment. Successful fractionation was achieved and resulted in clean separation of U1 small nuclear RNA from lysine-tRNA (Fig. 6). At 5 hpi, pre-miR-138-1 was found to be present at equivalent levels in the two fractions. However, at 12 hpi, there was much more pre-miR-138-1 in the nuclear fraction than in the cytoplasmic fraction, indicating inefficient nuclear export after 5 hpi. Also, much larger fractions of pre-miR-H1 and -H6 were found in the nucleus than in the cytoplasm at 12 hpi (Fig. 6). Similar results were observed with pre-miR-H6 and mature miR-H6 in WT-infected cells (Fig. S4). We found no evidence of impaired Dicer cleavage as judged by the relatively low pre/mature ratios in the cytoplasmic fractions at 5 hpi for miR-138 and at 12 hpi for both miR-138 and miR-H6 (Fig. 6; see also Fig. S4). Therefore, we conclude that HSV-1 infection impedes miRNA biogenesis by blocking nuclear export.

10.1128/mBio.02856-18.4 FIG S4 Nuclear export of pre-miR-H6 is blocked at late times during HSV-1 lytic infection. 293T cells were mock infected or infected with HSV-1 strain KOS for 5 or 12 h, and cells were harvested at both times for either direct RNA purification (total) or for separation into nuclear and cytoplasmic fractions prior to RNA purification. The resulting RNAs were subjected to Northern blot hybridization, probing for the RNAs indicated to the right of the panels. Mock infection times and times postinfection are indicated at the top of the panel. The fractions are indicated above the images. T, total RNA; N, nuclear fraction; C, cytoplasmic fraction. This experiment was performed multiple times. Similar results were obtained in an experiment using WTLyt138 virus (Fig. 6). Download FIG S4, PDF file, 0.3 MB. Copyright © 2019 Pan et al. 2019 Pan et al. This content is distributed under the terms of the Creative Commons Attribution 4.0 International license.

### ICP27 and viral DNA synthesis but not Us11 are necessary for blocking pre-miRNA processing.

**FIG 7:**
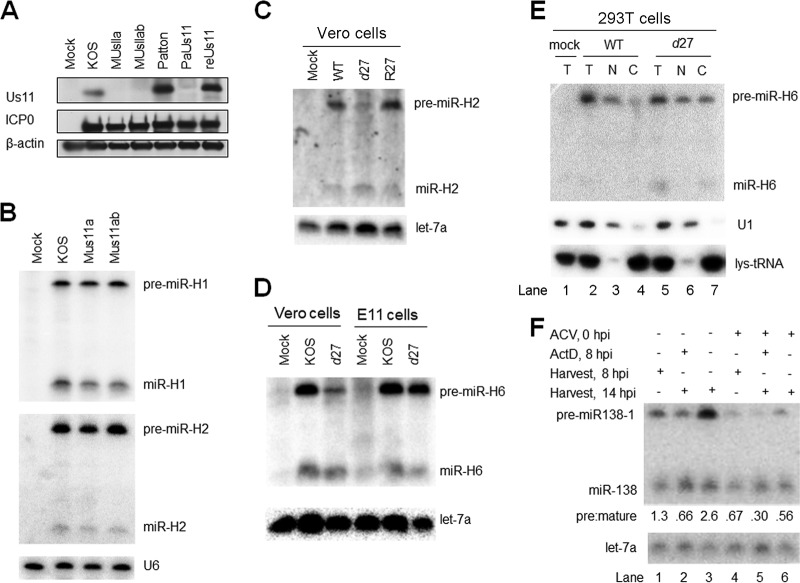
Impairment of miRNA biogenesis is dependent on ICP27 and viral DNA synthesis but not on Us11. (A) Us11 expression from WT and mutant viruses. 293T cells were infected with the viruses indicated at the top of the panel (MOI = 5) and harvested at 16 hpi for Western blot analysis. The proteins analyzed are identified to the left of the panel. This experiment was performed three times. (B) *Us11* deletion does not affect viral miRNA biogenesis. 293T cells were infected (MOI = 5) with the viruses indicated at the top of the panel. At 16 hpi, cells were harvested and analyzed by Northern blot hybridization for miR-H1 and miR-H2 expression. This experiment was performed several times. (C) Effects of *ICP27* deletion on miR-H2 biogenesis. Vero cells were mock infected or infected (MOI = 20) for 24 h with the viruses indicated at the top of the panel. Pre-miR-H2 and miR-H2 expression (top panel) and let-7a expression (bottom) were analyzed by Northern blot hybridization. This experiment was performed twice. (D) Effects of *ICP27* deletion on miR-H6 biogenesis in Vero and E11 cells. Vero cells, or E11 cells, which complement ICP27 expression, were infected with the viruses indicated at the top of the panel (MOI = 20) for 24 h, when the cells were analyzed by Northern blot hybridization for miR-H6. Expression of let-7a as a loading control is shown at the bottom. This experiment was performed twice. (E) Relief of the block of nuclear export in the absence of ICP27. 293T cells were mock infected (lane 1) or infected with the viruses indicated at the top of the panel. At 12 hpi, total RNA was isolated from infected cells (T; lanes 1, 2, and 5) or from nuclear (N; lanes 3 and 6) or cytoplasmic (C; lanes 4 and 7) fractions and analyzed as described for Fig. 6 for miR-H6, U1, and lys-tRNA (as indicated to the right of the panel). This experiment was performed twice. (F) Effects of ACV and ActD on miRNA biogenesis. 293T cells were infected (MOI = 5) with WT-BAC (lane 1) as a negative control for miR-138 expression or with WTLyt138 (lanes 2 to 7), as indicated in the top two lines of the panel. Plus signs (+) on the next two lines indicate treatment with ACV at the time of infection (lanes 5 to 7) or with ActD at 8 hpi (lanes 3 and 6), respectively. ACV was added to block viral DNA synthesis, and ActD was added to block RNA synthesis to help assess the stability of pre-miR-138 and mature miR-138. Plus signs on the next two lines indicate whether infected cells were harvested for RNA isolation at 8 hpi (lanes 2 and 5) or 14 hpi (lanes 1, 3, 4, 6, and 7). The image below shows the results of Northern analysis of expression of pre-miR-138-1 and miR-138, as indicated to the left. Pre/mature ratios calculated following band quantification are displayed below the image. let-7a levels as loading controls are shown at the bottom. This experiment was performed twice.

Hypothesizing that viral gene products might be involved in impeding miRNA biogenesis, we first tested the idea that Us11, an RNA binding protein with affinity for double-stranded RNA and even higher affinity for imperfect stem-loops (29, 30), might sequester pre-miRNAs, especially as Us11 has been suggested to block RNA silencing (31). However, pAUs11 mutant virus derived from strain Patton (32), which is Us11-defective due to a polyadenylation signal 5′ to the *Us11* start codon, showed pre/mature ratios for miR-H1 and miR-H2 similar to those of the corresponding rescued virus (data not shown). Because we detected a weak immunoreactive band that comigrated with strain Patton Us11 in pAUs11-infected cells on a Western blot (Fig. 7A), we wondered whether leaky expression might be sufficient to impede miRNA biogenesis. We therefore constructed two additional Us11 mutant viruses on the basis of our KOS-derived WT-BAC virus (33). One virus, named MUs11a, removes an A from the *Us11* start codon, which should result in translation starting from an out-of-frame AUG. The other virus, named MUs11ab, further replaces the second and third in-frame ATG with TGA stop codons in the MUs11a virus. Ablation of Us11 immunoreactivity comigrating with KOS Us11 was confirmed for both viruses (Fig. 7A). Both viruses exhibited levels of pre-miR-H1 and pre-miR-H2 and mature miR-H1 and miR-H2 similar to those seen with the WT (Fig. 7B), suggesting that Us11 is dispensable for regulation of miRNA biogenesis.

Another candidate for impeding miRNA biogenesis is ICP27, which plays a role in mRNA nuclear export (reviewed in reference 34). A homolog of ICP27 has been reported to block RNA silencing in plants (35). To test its involvement in miRNA biogenesis, we utilized an *ICP27*-null virus, *d*27 derived from HSV-1 strain KOS1.1 (36). Following infection of Vero cells, *d*27 expressed similar or possibly greater amounts of mature miR-H2 but substantially lower amounts of pre-miR-H2 than did both its parental WT virus and the rescued derivative, R27 (Fig. 7C). In another experiment, *d*27 expressed amounts of mature miR-H6 similar to those seen with the WT virus but much less pre-miR-H6 than the WT virus in infected Vero cells, and yet the *d*27 and WT viruses expressed similar amounts of pre-miR-H6 and mature miR-H6 in E11 cells, which ectopically express ICP4 and ICP27 (37) (Fig. 7D). In a third experiment, total RNA or RNA from nuclear and cytoplasmic fractions was isolated from WT and *d27*-infected 293T cells at 12 hpi. At that time point, similar to the results seen at the 12-hpi point (Fig. 6), WT virus expressed readily detectable pre-miR-H6 but much less mature miR-H6 (Fig. 7E, lane 2). The pre-miR-H6 was primarily found in the nuclear fraction (lane 3), with substantially less seen in the cytoplasmic fraction, and that small amount could be accounted for by nuclear contamination as evidenced by detection of U1 snRNA (lane 4). In contrast, the ratio of pre-miR-H6 to mature miR-H6 was lower in *d27*-infected cells than in WT-infected cells at 12 hpi (compare lanes 5 and 1), and the levels of pre-miR-H6 were similar in the nuclear and cytoplasmic fractions, which were cleanly separated based on the U1 and lys-tRNA markers. Taken together, these results show that ICP27 contributes to high pre/mature ratios and to retention of pre-miRNA in the nucleus.

Finally, we tested whether the impairment in miRNA biogenesis is dependent on viral DNA synthesis. 293T cells were infected with WTLyt138 in the absence or presence of acyclovir (ACV), an inhibitor of HSV DNA synthesis. At 8 hpi, infected cells were either mock treated or treated with ActD, and RNA was harvested immediately or at 14 hpi. In the absence of ACV and ActD, the miR-138 pre/mature ratios increased between 8 and 14 hpi (Fig. 7F, lanes 2 and 4), similarly to what we observed in Fig. 5D. Adding ActD in the absence of ACV at 8 hpi resulted in little change in pre-miR138-1 and mature miR-138 levels between 8 hpi and 14 hpi (lanes 2 and 3), consistent with the data in Fig. 4D indicating inefficient conversion of pre-miRNA to mature miRNA. However, in the presence of ACV, at both 8 and 14 hpi, the levels of mature miR-138 were similar to those seen in the absence of ACV at the same time points, but the levels of pre-miR-138-1 were considerably lower (compare lanes 5 and 7 with lanes 2 and 4), similarly to what we observed in the absence of ICP27 (Fig. 7C to E). Addition of ActD to ACV-treated infected cells at 8 hpi resulted in a decrease in pre-miR-138 and an increase in mature miR-138 (compare lanes 5 and 6), indicating conversion of pre-miRNA to mature miRNA. Thus, high pre/mature ratios are dependent on both ICP27 and viral DNA synthesis but not on Us11.

## DISCUSSION

By characterizing HSV-1 pre-miRNA and miRNA expression in transduced and infected cells and in mouse ganglia, we found that although these miRNAs differed in inherent biogenesis efficiencies, conversion of pre-miRNA to miRNA was considerably less efficient during lytic infection than during latent infection or following lentiviral transduction, regardless of the miRNAs or cell types tested. Much if not all of this impairment of miRNA biogenesis was due to a block in pre-miRNA export from the nucleus to the cytoplasm. High pre/mature ratios occurred late in infection and were dependent on ICP27 and viral DNA synthesis but not on Us11. Below, we discuss these results and their implications for HSV-1 infection.

### Regulation of miRNA biogenesis during HSV-1 lytic infection and variability in intrinsic biogenesis efficiency both contribute to miRNA expression.

Our Northern results show that all HSV-1 miRNAs tested exhibited high pre/mature ratios during lytic infection of multiple cell lines. Consistent with this, most exhibited high pre/mature ratios in ganglia at 3 dpi, arguing for a regulatory mechanism that applies to both acute ganglionic infection and lytic infection of cultured cells. During latency, pre/mature ratios were considerably reduced, arguing that miRNA biogenesis became more efficient, presumably due to reduced expression of the viral function(s) that impaired miRNA biogenesis. This mechanism then likely contributed to high expression of these miRNAs during latency.

Based on our results from lentivirus-transduced cells, the order of intrinsic efficiencies of conversion of pre-miRNA to miRNA is miR-H4 > miR-H2 > miR-H3, consistent with the relative pre/mature ratios for these miRNAs in infected cells and ganglia and suggesting that intrinsic biogenesis efficiencies contribute to determining expression levels. The finding of different intrinsic biogenesis efficiencies explains the inability to detect mature miR-H3 during lytic or latent infection by Northern analysis and the inability to detect pre-miR-H4 in infected ganglia. Variability in intrinsic biogenesis efficiency may reflect sequence-specific differences in pre-miRNA export and/or Dicer cleavage (38, 39). Regardless, for each miRNA tested, including the host miR-138, pre-miRNA conversion to miRNA was more efficient in lentivirus-transduced cells than in HSV-1 lytically infected cells, consistent with impairment of miRNA biogenesis during lytic infection.

### Mechanism of impairment of miRNA biogenesis during lytic infection.

miRNA biogenesis during lytic infection of cells is temporally regulated. When detectable at early times of infection (e.g., in the case of ectopically expressed miR-138), pre-miRNAs convert efficiently to miRNAs, but the level of conversion is reduced later in infection. Moreover, pre-miRNAs exhibit short half-lives in the presence of ActD at 2 hpi but are much more stable at 8 hpi. Thus, impairment of miRNA biogenesis is a late infection event. Consistent with this, ACV treatment resulted in both reduced pre/mature ratios and reduced stability of pre-miRNA in the presence of ActD. Similarly, our finding that pre-miRNAs were depleted in the cytoplasm and enriched in the nucleus at 12 hpi, but not at 5 hpi, indicates that inhibition of nuclear export of pre-miRNAs occurs late. Thus, the results strongly suggest that inhibition of nuclear export of pre-miRNAs explains the impairment of miRNA biogenesis late during lytic infection. However, we cannot exclude the possibility of effects on other steps. Blocking nuclear export of pre-miRNAs is likely not unique to HSV-1 infection. Baculovirus encodes a miRNA that was reported to target the Exportin-5 cofactor Ran (40). Accumulation of pre-miR-31 in the nucleus with undetectable mature miR-31 has been reported in certain cancer cell lines (41), while activation of the oncogenic protein kinase extracellular signal-regulated kinase (ERK) has been reported to downregulate nuclear export of pre-miRNAs in hepatocellular carcinoma cells (42). Understanding how HSV blocks nuclear export of pre-miRNAs may reveal mechanisms that regulate miRNA biogenesis in other viral and cellular systems.

Deletion of *ICP27* also greatly reduced pre/mature ratios for HSV-1 miRNAs. Interestingly, this deletion reduced the expression of the pre-miRNAs with no reduction or possibly an increase in the levels of the mature miRNAs, which we suggest was due to these pre-miRNAs being late viral gene products (12) and thus likely to be dependent on ICP27 for their full expression. Nevertheless, the simplest interpretation is that ICP27, one of whose many functions (34) is to promote export of certain mRNAs (43–45), is required for impairment of miRNA biogenesis. Assuming that this is correct, the issue becomes whether ICP27 itself acts to impair miRNA biogenesis or whether ICP27 is required for synthesis of one or more viral gene products that do so. Supporting the former possibility, the turkey herpesvirus (HVT) homolog of ICP27 has been reported to be sufficient for blocking RNA silencing in tobacco (35), and it has been speculated that ICP27 may block nuclear export of certain RNAs (46). Supporting the latter possibility are the immediate early expression of ICP27 versus the late impairment of miRNA biogenesis and the similar phenotypes of ACV treatment and *ICP27* deletion, especially given that most viral late and certain DNA replication proteins require ICP27 for their efficient synthesis (47). A third possibility is that HSV-1 induces expression of a host factor that impairs miRNA biogenesis in an ICP27-dependent fashion. Experiments are under way to test these alternatives.

### Implications for HSV-1 infection.

**FIG 8:**
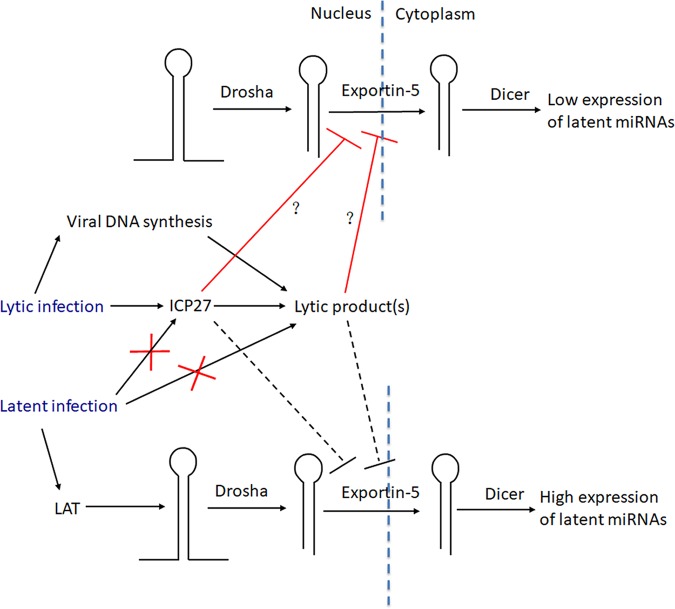
Cartoon of proposed mechanism of regulation of miRNA biogenesis during HSV-1 infection. The cartoon is divided into a nuclear portion to the left and a cytoplasmic portion to the right, with vertical dotted lines representing the nuclear membrane. The upper and lower parts of the cartoon illustrate events occurring during lytic infection and latent infection, respectively. Pre-miRNA stem-loop structures are represented by hairpins. Lines terminating with arrowheads point from causes to results of events, with important factors shown above the arrows and with each red X indicating a loss of expression of important factors. Red lines terminating with perpendicular lines indicate events being blocked by ICP27 and/or lytic products dependent upon ICP27 for their synthesis, with the dashed line indicating a loss of the block.

Our results lead to a model (Fig. 8) in which HSV-1 lytic infection blocks pre-miRNA conversion to miRNAs at the stage of nuclear export whereas lytic gene products responsible for this block are depleted during latent infection, allowing more efficient biogenesis. Certain HSV miRNAs that are expressed abundantly during latency have been shown to be able to repress expression of important lytic proteins such as ICP0, ICP4, and ICP34.5 in transfection experiments (3, 9, 10, 13–15). miRNAs that are abundant during latency might also inhibit the expression of host proteins that would otherwise promote lytic rather than latent infection. Thus, latent HSV miRNAs have been hypothesized to promote latency. Although some *in vivo* mutational studies of HSV-1 and HSV-2 miRNAs have yielded negative results (15, 25), interpretation of these results is complicated by the possibility of functional redundancy and the limitations of using small animal models to recapitulate latency in humans using small-animal models. Blocking miRNA biogenesis during lytic infection may serve to limit the extent to which expression of their targets is repressed, allowing less-restricted viral replication. This proposal is supported by a study that showed that HSV-1 infection suppresses RNA-induced silencing and that knockdown of Ago2 expression increases HSV-1 replication (48). During latent infection, when repression of lytic gene expression (or certain host factors) is beneficial to the virus, removal of the block to miRNA biogenesis combined with high transcription of the *LAT* locus permits strong expression of latent miRNAs (Fig. 8).

In plants and invertebrates, viruses rely on virus-encoded suppressors of RNA interference (RNAi) to combat an RNAi-mediated antiviral response, although the functional relevance of this suppressive activity in mammals remains to be established (49). Mammalian viruses, including some herpesviruses, have displayed various ways of regulating miRNA biogenesis (reviewed in references 19 and 50). However, we are unaware of any previous examples of regulation of pre-miRNA nuclear export by mammalian viruses. Effects of viral infection on expression of regulators of miRNA biogenesis have been observed with gammaherpesviruses (see, e.g., references 51 and 52), and there is evidence that levels of certain mature viral miRNAs are regulated posttranscriptionally (see, e.g., references 53 and 54). Specific downregulation of a host miRNA by overexpression of a complementary target has been reported previously for both betaherpesviruses and gammaherpesviruses (55–58). However, although the high pre/mature ratios of most viral miRNAs that we document here can also be found during lytic infection with HSV-2 and pseudorabies (9, 10, 24, 25, 59), they have not been generally observed during the lytic cycles of the avian alphaherpesvirus, Marek’s disease virus, betaherpesviruses, or gammaherpesviruses (60–66). Therefore, general impairment of conversion of pre-miRNA to mature miRNA appears to be found mainly among mammalian alphaherpesviruses. We speculate that this might relate to their neurotropism. Regardless, our findings identify a novel mechanism of differential regulation of miRNA expression that may ensure optimal outcomes for lytic and latent infection.

## MATERIALS AND METHODS

### Cells and viruses.

Vero, 293T, and Neuro-2A cells were maintained as previously described (28). Vero and Neuro-2A cells were obtained from ATCC and used following less than 6 months of passage. 293T cells were authenticated by small-tandem-repeat profiling at the Dana-Farber Cancer Institute Molecular Diagnostics Laboratory in December 2018. Viruses were propagated in Vero cells and titrated by plaque assays following standard protocols (67). WTLyt138, MUs11a, and MUs11b viruses were constructed based on a BAC-containing strain, KOS (33), by two-step red recombination (68). To construct WTLyt138 virus, the following human pre-miR-138-1 and flanking sequences were synthesized as a gBlocks gene fragment (Integrated DNA Technologies): CTTAGAATTCGGATCATCAATCATCTGCCTTCCCAGGACCACCCAGCAGTGTTGCACGTAGAGCAGAGGTGCAGTGCTGCACGTACAGCAGAGGCGACCCCTTCTCCATACTTCAGAGACCTCTAGCATCGTGTTGTGGGACAGCTGGTGTTGTGAATCAGGCCGTTGCCAATCAGAGAACGGCTACTTCACAACACCAGGGCCACACTGCACTGCAAGCAGCGAGCAGCAAGCAACAAGCATCCAGACACCTGAGCACCAGGCTGCCAGCAAGAGCACCTGCCTGTGAGGAAACCTCCCAGCCCACCCCATACCCCACCCCATTATCCCACCCTACAAGCTTGGTA. The sequences were cloned between the EcoRI and HindIII sites of FLAG-HA-pcDNA3.1(-) plasmid (AddGene). A kanamycin resistance gene (*Kan*) flanked by one I-SceI site was then cloned into the EcoRI site of the resulting plasmid using the following two primers: primer CTTAGAATTCGGATCATCAATCATCTGCCTTCCCAGGACCACCCAGCAGTGTTGCACGTATAGGGATAACAGGGTAATCGATTT and primer CTTAGAATTCGCCAGTGTTACAACCAATTAACC. The resulting plasmid was used as a template to amplify a cassette to be recombined with the HSV-1 genome using the following two primers: primer AATCCGGTAACCCGTTGAGTCCCGGGTACGACCATCACCCGAGTCTCTGGAATTCGGATCATCAATCATCTGCC and primer GGGTTGGGTCTGGCTCATCTCGAGAGACACGGGGGGGAACCACCCTCCGCGTAGGGTGGGATAATGGGGTGG. The subsequent recombination of this cassette with the viral genome and removal of *Kan* were conducted as described previously (68). The two-step red recombination protocol was also used to construct MUs11a BAC using primer TACGACCATCACCCGAGTCTCTGGGCGGAGGGTGGTTCCCCCCCGTGTCTCTCGAGTGAGCCAGACCCAAcCCCCGTAGGGATAACAGGGTAATCGATTT and primer TAAGTAAACATCTGGGTCGCCCGGCCCAACTGGGGCCGGGGGTTGGGTCTGGCTCACTCGAGAGACACGGGGGGGAGCCAGTGTTACAACCAATTAACC as well as to construct MUs11b based on MUs11a using primer TGTTTACTTAAAAGGCGTGCCGTCCGCCGGCTGACACCCCAGAGGTGTTCACGCACCTCGAGGACACCCGCGCTGATAGGGATAACAGGGTAATCGATTT and primer TCATTATCACCCCGTTGCGGGGGTCCGgAGATTCAGCGCGGGTGTCCTCGAGGTGCGTGAACACCTCTGGGGTGTCAGCCAGTGTTACAACCAATTAACC.

Viruses were produced by transfecting BAC DNAs into Vero cells using Lipofectamine 3000 following the instructions of the manufacturer (Thermo Fisher). Cells were infected by adding virus at the indicated MOI, incubating for 1 h at 37°C with gentle shaking every 15 min for adsorption, and replacing the supernatant with fresh media and incubating at 37°C for the indicated times.

### Northern blot hybridization.

RNAs of <200 nucleotides were purified from cultured cells or pooled mouse ganglia using a miRNeasy minikit (Qiagen) following the manufacturer’s instructions and were resolved on Novex 15% Tris-borate-EDTA-urea polyacrylamide gels (Thermo Fisher). We used radioactive and nonradioactive methods interchangeably for Northern blot hybridization. The nonradioactive method followed a previously described protocol (15). LNA probes were synthesized and 3′ end labeled with digoxigenin by Exiqon. The radioactive method followed the same protocol before hybridization. During the prehybridization step, 10 pmol of probe was incubated with 1 μl of T4 polynucleotide kinase (NEB), 2.5 μl of the supplied buffer, and 1 to 5 μl of [γ-^32^P]ATP (Perkin Elmer) in a total volume of 25 μl for 1 h at 37°C before this mixture was added into the hybridization tube. The subsequent hybridization and washing conditions were the same as those used with the nonradioactive method. After the last wash, the membrane was exposed to a phosphor screen and visualized by a phosphorimager. Band intensities were quantified by Quantity One 1-D Analysis Software (Bio-Rad). The probe sequences and optimized hybridization and washing temperatures for different RNAs are listed in Table 1.

**TABLE 1:** Sequences of Northern blot probes

Probe designation	Probe sequence^*a*^	Optimized hybridization and washing temp (°C)^*b*^
miR-H1	TCCACTTC+CC+G+TC+C+TTCCATC	60
miR-H2	AGTCGC+AC+TCG+T+CCCTGGCTCAGG	60
miR-H3	GTCCCA+A+C+C+G+C+A+C+A+GTCCCAG	65
miR-H4	ACTAGC+G+AG+TT+AGA+C+AGGCAAG	55
miR-H5	AGGG+TT+T+GG+ATC+T+CTGAC	60
miR-H6	GGGA+TG+GA+AG+GA+CGGG+AAGTG	55
miR-138	CGGCCTG+A+TT+C+A+CA+ACACCAGCT	60
let-7a	AACTAT+A+CA+A+CCT+A+CTACCTCA	60
U6	CGAAT+TTG+CGT+GT+CAT+CCTTGC	65
U1	CGAGTTTCCCACATTTGGGG	37
lys-tRNA	CTGATGCTCTACCGACTGAGCTATCCGGGC	37

a The probes are labeled with digoxigenin at the 3’ end, for nonradioactive detection. +N, locked nucleic acid-modified nucleotide.

b The washing temperature is typically the same as hybridization temperature, but during the last two washes, raising the temperature by 5°C helps further remove nonspecific bands.

### Animal procedures.

The animal housing and experimental procedures were approved by the Institutional Animal Care and Use Committee of Harvard Medical School in accordance with federal guidelines. Male CD-1 mice (Charles River Laboratories) (7 weeks old) were infected with HSV-1 strain KOS as described previously (15). At the indicated times, trigeminal ganglia were removed and placed on dry ice before being stored at −80°C. For reactivation, trigeminal ganglia removed from mice at 30 dpi were held in Dulbecco’s modified Eagle medium supplemented with 10% fetal bovine serum on ice during the dissection procedure and were then incubated at 37°C for 1 to 3 days before the ganglia were transferred to Eppendorf tubes. Sixteen ganglia were pooled for each condition before RNA purification.

### Construction of lentivirus-transduced cell lines.

The synthesized gBlocks gene fragment described above was used as a miR-138-expressing sequence. miR-H2-expressing and miR-H3/H4-expressing sequences were amplified from HSV-1 WT BAC using primers TGACCTCGAGTTTTCCTGGCCCGACCCGCGCCTCTT and CTGTGAATTCGACCCCATAGTGATCAGCGACTCC for miR-H2 and primers TGACCTCGAGGACGCGGACTCGGGAACGTGGA and CTGTGAATTCTCGCGCGCGGCCCTTTAAAGGCG for miR-H3/H4. Each miRNA-expressing sequence was inserted between the XhoI and EcoRI restriction sites of pTRIPZ (69) (a generous gift from Bryan Cullen). A 4-μg amount of each of the resulting plasmids was transfected together with 7.1 μg of psPAX2 plasmid (Addgene) and 3.9 μg of pMD2.G plasmid (Addgene) into 293T cells in a 100-mm-diameter plate using Lipofectamine 3000 following the instructions of the manufacturer (Thermo Fisher). Lentiviruses were harvested from the supernatants 3 days posttransfection and added to 50% confluent 293T or Neuro-2a cells in the presence of 8 μg/ml of hexadimethrine bromide (Sigma). Two days later, the supernatant was removed and replaced with fresh medium containing 1 μg/ml of puromycin. Surviving cells were expanded in the presence of puromycin until the cells were stably maintained.

### Western blotting.

Western blotting was performed as described previously (57). The following primary antibodies (manufacturer and catalog number or reference number) were used at the indicated dilutions: ICP0 antibody (Abcam, ab6513), 1:5,000; ICP27 antibody (Virusys, 1113), 1:5,000; β-actin antibody (Sigma, A5441), 1:10,000; Drosha antibody (Abcam, ab12286), 1:1,000; Dicer antibody (Santa Cruz, H212), 1:200; Exportin 5 antibody (Santa Cruz, H-300), 1:200; Us11 antibody (70), 1:1,000.

### Cell fractionation.

Cell fractionation was performed using a tissue grinder (Wheaton) (1 ml) as previously described (71). RNA was purified from total, cytoplasmic, and nuclear fractions using a mirVana miRNA isolation kit following the instructions of the manufacturer (Ambion).
