# Supplementary figures and images for: Herpes Simplex Virus 1 Lytic Infection Blocks MicroRNA (miRNA) Biogenesis at the Stage of Nuclear Export of Pre-miRNAs
Source: mBio. 2019 Feb 12;10(1):e02856-18. doi: 10.1128/mBio.02856-18 (PMC6372804; doi:10.1128/mBio.02856-18)

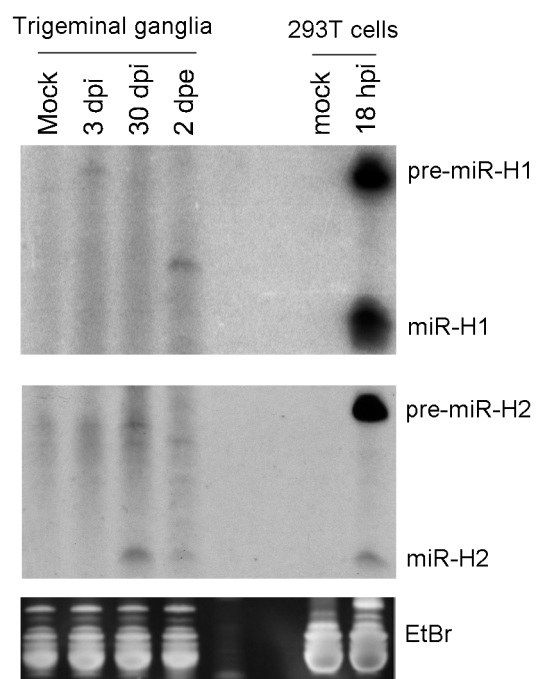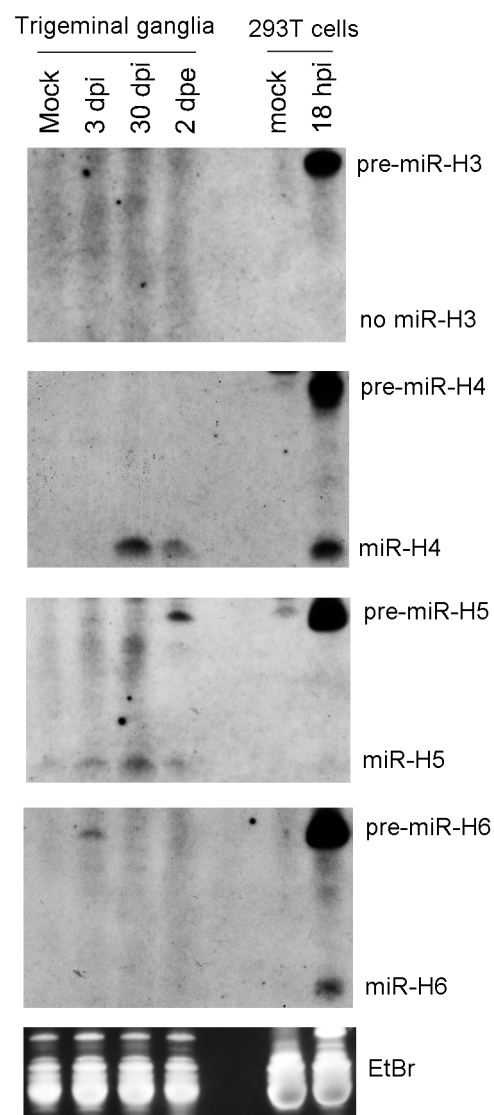

Supplement: FIG S1 [file mBio.02856-18-sf001.pdf]

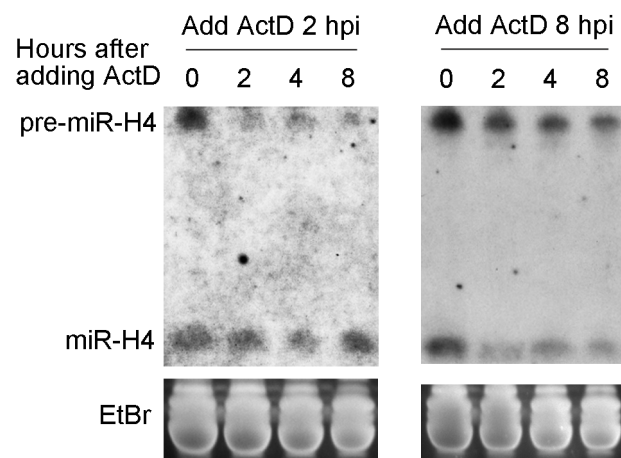

Supplement: FIG S2 [file mBio.02856-18-sf002.pdf]

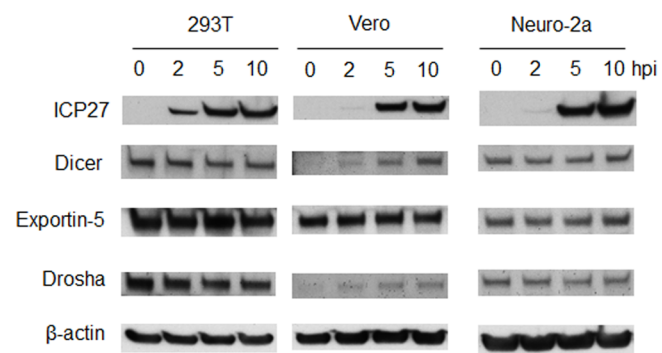

Supplement: FIG S3 [file mBio.02856-18-sf003.pdf]

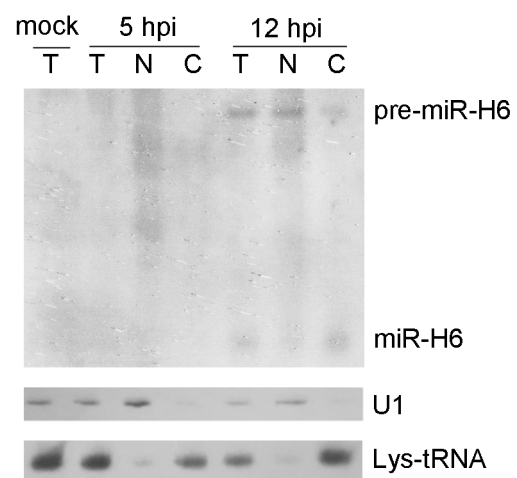

Supplement: FIG S4 [file mBio.02856-18-sf004.pdf]
